# Certified Nursing Aides' Training Hours and COVID Case and Mortality Rates Across States in the U.S.: Implications for Infection Prevention and Control and Relationships With Nursing Home Residents

**DOI:** 10.3389/fpubh.2022.798779

**Published:** 2022-04-08

**Authors:** Lené Levy-Storms, Amelia Mueller-Williams

**Affiliations:** ^1^Geffen School of Medicine, Department of Medicine, Division of Geriatrics, Los Angeles, CA, United States; ^2^Department of Social Welfare, Luskin School of Public Affairs, University of California, Los Angeles, Los Angeles, CA, United States

**Keywords:** nursing aides, nursing homes, COVID, training, communication, CNAs, policy, pandemic

## Abstract

Disproportionately high COVID case and mortality rates in skilled nursing facilities (SNFs) have heightened interest in the role of Certified Nursing Aides (CNAs) in the care of residents living in SNFs. This policy brief will make recommendations for CNA training based on an examination of two sources of secondary data using descriptive statistics. From the first source of secondary data, 34% of CNAs report feeling inadequately trained. The second source, U.S. government data, revealed statistically significant negative correlations between the amount of CNA training required across states and COVID mortality rates (Kendall's τ_b_ = −0.32; *p* = 0.002) but not case rates (Kendall's τ_b_ = −0.18; *p* = 0.09). More training for CNAs may not only reduce health risks from infectious diseases but also improve how they relate to SNF residents during care.

## Introduction

In the U.S., COVID-19 exposed multiple vulnerable social strata one of whom was the “oldest old” among adults living in skilled nursing facilities (SNFs). The fastest growing group of older adults is 85 years and older (1). Close to half of SNF residents are over 85 years (2), and the average age of residents is in the 80s (3). They had one of the highest mortality rates at the beginning of the pandemic, and high infection rates soon followed among their paid caregivers (3, 4). These paid caregivers, certified nursing aides (CNAs), provide 90% of direct care to SNF residents (5), thus, providing a possible basis for the connection between high mortality and infection rates among SNF residents. SNFs faced increased scrutiny for these high mortality and case rates that specifically noted their lack of personal protective equipment (PPE) and its inconsistent availability and use among staff. While the benefits of PPE have monumental impact in reducing the spread of COVID-19 and mitigating mortality when available and used correctly, how other aspects of CNAs' training may associate with the spread and consequences of the pandemic remain unclear.

Similar issues occurred across the world, but the strength of the connection between infection rates among SNFs' (or equivalent care facilities) staffs and residents and their mortality rates varied (6). This may be, in part, due to multiple (e.g., size of facilities, safety regulations and resources, ventilation, etc.) across countries including PPE availability and use among SNFs' staffs (4, 7–11). Another possible factor may include how well trained these staffs were in infection prevention and control (IPC). In the U.S., publically-available information can provide data as a case study for how infection and mortality rates from the Centers for Disease Control and Prevention (CDC-P) vary by training. Federal data exist for states' variation in training but less so for data on PPE and other resources (12, 13). In the U.S., federal policies establish a minimum on CNA training hours, but state policies vary in how far they go beyond the these minimums, if at all.

This policy brief will examine CNAs' training policy options based on publically-available, secondary data from different departments of the U.S. Government and the Paraprofessional Healthcare Institute (PHI). One data source comes from a nationally-representative survey of CNAs' perceptions of the quality of their initial and ongoing training. Another publically-available secondary data source includes state variation in training hours as well as COVID mortality and case rates among SNFs' residents. In this policy brief, the investigation into CNAs' training perceptions, hours and SNFs' residents' mortality and case rates from COVID will have implications for how CNAs' training affects their social interaction and care for SNFs' residents. Such paid caregiving is a type of formal social relationship and like other social relationships has implications for residents' physical health and overall wellbeing (14).

## Policy Options and Implications

### Certified Nursing Aides Compared to Other Direct Care Workers

Certified nursing aides or CNAs compose one type of direct care worker, which includes home health and residential aides. All of these direct care workers provide primarily custodial care for activities of daily living (ADLs, e.g., eating, dressing, etc.) and sometimes instrumental activities of daily living (IADLs, e.g., light house cleaning, cooking, etc.) (15). CNAs represent relatively the smallest subgroup of direct care workers, composing only 12% (15), but their certification makes them impactful since only CNAs can care for SNFs' residents. While CNAs share the provision of ADLs with other direct care workers, CNAs differ from the other type of direct care workers based on their certification. While CNAs may work in home health agencies or assisted living along with uncertified nursing aides, in SNFs they have to be certified. Any SNFs' that receive federal reimbursements must employ only CNAs, based on the Nursing Home Reform Act of 1987 (12). In this legislation, certification requires nursing aides to undergo a minimum of 75 h of combined classroom and clinical training on the scope of direct care for SNFs' residents including feeding, dressing, and bathing, for example, as well as safety protocols such as how to lift residents correctly without injury and environmental management like changing bed pans. States may have additional training beyond this federal minimum, and some do. Regardless, regulatory requirements for CNAs can set the standards for other direct care workers should non-certified direct care workers become more regulated in the future. Regulations can have an even larger impact in the face of pandemics like COVID, since CNAs have to abide by them but other direct care workers may not. For example, President Biden required all CNAs to be vaccinated against COVID (16).

Federal requirements dictate the scope and total hours of training but they do not provide specific curricular content, assessments, or detailed protocols for clinical training. No universally-accepted training standards exist (15). The Affordable Care Act of 2010 (17) did introduce a dementia care training requirement but left it to SNFs' to decide what curriculum to use. Consequently, many variations on dementia care training exist, including an optional one from the Centers for Medicare and Medicaid (CMS) (18). Clinical hours, also called supervised practical hours, must cover five content areas including infection control and communication with residents, for example (13). CNAs may receive training for certification from a number of sources including their SNFs' of employment, community college, Red Cross site, or nursing school (19, 20). Online certification is also offered for the classroom portion of their training. Regardless of the source of training, no federal requirements dictate how to cover these areas and do not include universally-accepted competencies like other professional licensure programs. Inconsistencies and gaps across curricula for CNA certification weaken existing regulations, because while current regulations form the structure (e.g., hours, scope of content, etc.) for quality care, they fall short of ensuring the process (e.g., competencies/skills, provision of care, etc.) of quality care. Despite the ACA's addition of dementia care to training requirements, substantive improvements remain in need to address inconsistencies and gaps in curricular content for CNA training.

The first and only nationally-representative survey of specifically CNAs occurred in 2004 under the U.S. Department of Health and Human Services. CNAs responded to questions about their training and its adequacy. From this survey, 34% of CNAs reported feeling inadequately trained initially and 25% reported that continuing education classes were only “somewhat to not at all useful” (21, 22). Since this survey occurred over 17 years ago, a 2019 Census survey provides a more current reference point for demographics. Table 1 shows both 2004 demographic data on CNAs and U.S. Census data on CNAs from 2019 that revealed trends such as CNAs from 2019 were younger, less educated, more ethnically diverse, less likely to be married, higher paid and more full-time employment. Otherwise, data on CNAs from these two different but nationally-representative surveys indicated similar levels of median income and percent female. The trends in these demographic data combined with the percent of CNAs feeling inadequately trained suggest that CNAs today may feel even less well-trained, given their lower education in 2019 compared to 2004.

**Table 1:** Demographic characteristics of certified nursing aides (CNAs) in 2004 compared to 2019.

**Demographic characteristics of CNAs**	**2004^**a**^**	**2019^**b**^**
	**%/$**	**%/$**
Age (median)	39.5^1^	37
Female (%)	92.0	91.0
Education (%)		
Less than high school	12.4	9.0
Race/Ethnicity (%)^c^	53.4	42.0
White	38.7	38.0
Black	7.9	20.0
Other		
Married (%)	50.7	36
Income		
Median in $	25,000^2^	24,200
Average hourly wage ($)	10.36	14.07
Full-time (%)	51.6	77.0
*N*	702,500	525,766

1 *Midpoint between 35–44 years*.

2 *Midpoint between $20,000 to < $30,000; N = weighted sample size*.

a *National Nursing Aide Survey (NNAS) 2004–05*.

b *2019 1-Year Public Use Microdata Sample (PUMS) from the American Community Survey (ACS)*.

c *Race/ethnicity measures between the NNAS and PUMS differ in that NNAS data has ethnicity (i.e., Hispanic/Not Hispanic) in each race category, but the PUMS data confined Hispanic/Not Hispanic only to the “Other” race category across all races. Thus, the “other” category in the PUMS is larger than the NNAS category of “other”*.

### Federal and State Variation in CNAs' Training Requirements

Variation across states in implementing the aforementioned training requirements for CNAs may exacerbate the inconsistencies and gaps in their structure. While all states have to abide by the federal minimum of 75 hours, states with little or no additional training requirements suggest a lack of investment and value for these direct care workers. The Paraprofessional Healthcare Institute (PHI), a non-profit patient care advocacy group collects data on state-level CNAs' training requirements demonstrating the wide variability of training requirements across states (Table 2). From these descriptive data, Table 2 shows how state CNAs' training requirements range from only federal minimums (18 states) to a maximum of 180 total hours of training (as in ME). The averages of the total training and clinical hours across all states and the District of Columbia (DC) are 98 and 39 hours, respectively. Alaska, California and Missouri have the most of both total and clinical hours and the highest percentages of clinical to total hours of all states and DC. In all, 35% of states and DC do not exceed the total federal minimum requirements.

**Table 2:** State- and DC-specific Certified Nursing Aide (CNA) training requirements.

	Number of states^*^	**States with requirements**
Only federal minimum hours (75 total, 16 SPT)	16^*^	AL, CO, KY, MA, MI, MN, MS, NE, NV, NM, NC, ND, OH, OK, SD, WY
Federal minimum total hours, supplemental SPT (75 total, >16 SPT)	3	IA, TN, VT
Exceeds federal minimums (>75 total, >16 SPT)	32	AK, AZ, AR, CA, CT, DE, DC, FL, GA, HI, ID, IL, IN, KS, LA, ME, MD, MO, NH, NJ, NY, OR, PA, RI, SC, TX, UT, VT, VA, WA, WV, WI

* *Two states, NV, DC and NM, did not have data on clinical hours available. The District of Columbia is included in these state-level data; N = 51*.

*Source: Paraprofessional Health Institute*.

### CNA Training Requirements and COVID-19 Mortality and Cases Among SNFs' Residents

**Figure 1:**
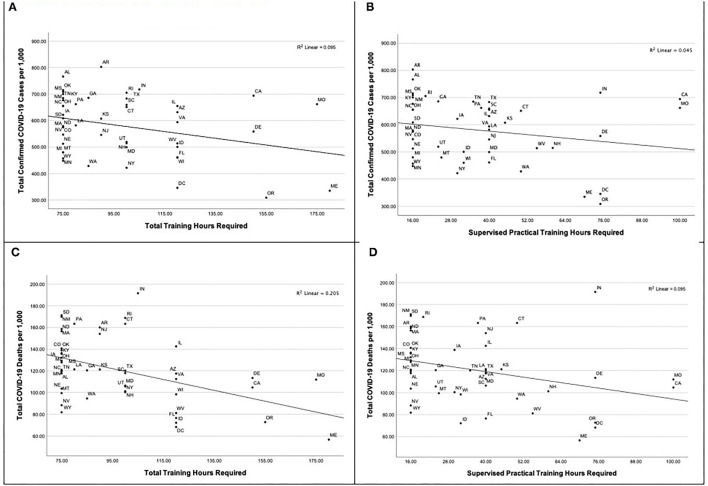
Association between State Training Requirements for Certified Nurisng Assistants and State Average Nuring Home Resident COVID-19 Infection and Death Rates, 01.01.2022 – 02.07.2021. **(A)** Association between state-required total training hours and nursing home resident COVID-19 infection rates, **(B)** Association between state-required supervised practical training hours and nursing home resident COVID-19 infection rates, **(C)** Association between state-required total training hours and nursing home resident COVID-19 death rates, **(D)** Association between state-required supervised practical training hours and nursing home resident COVID-19 death rates.

Additional secondary data exist on how CNA training across states associate with mortality and case rates. Table 3 has publically available data from two sources: PHI and the Centers for Disease Control and Prevention (CDC). Only trends will derive from this policy brief's correlational analyses. At the state level, descriptive trends based on Kendall's Tau for non-parametric data in Table 3 show a general negative association between COVID-19 mortality and case rates in SNFs' residents and state-level CNAs' training requirements. Overall, states with higher CNAs' training requirements tended to have lower COVID-19 mortality and case rates among SNFs' residents. Interestingly, the association between total hours of training is stronger and statistically significant for COVID-19 mortality rates (Kendall's τ_b_ = −0.32; *p* = 0.002) but not case rates (τ_b_ = −0.18; *p* = 0.09). The same trends and statistical significance occurred for total supervised practical hours (τ_b_ = −31;*p* = 0.003 for mortality rates and τ_b_ = −0.20; *p* = 0.05 for case rates). Figure 1 shows a graphical display of these correlations from this policy brief's correlational analyses. Thus, the potential protective effect of enhanced CNAs' training requirements may be more potent for preventing COVID-related deaths rather than COVID cases. Further research with controlled analysis could explore even better the relative contribution of CNAs' training to COVID-related deaths and cases in the presence of other predictive factors like size of facility, staffing ratios, and case mix of residents.

**Table 3:** Kendall's (τ_b_) correlations between state training requirements for certified nursing assistants (CNAs) state average COVID-19 infection and state death rates per 1,000 skilled nursing facilities' residents^*a*^.

	**Total training**	**Supervised practical training**
	**hours required**	**hours required**
Infection rate		
	τ_b_ −0.18	−0.20
	*p* = 0.09	*p* = 0.05
Death rate		
	τ_b_ −0.32	−0.31
	*p* = 0.002	*p* = 0.003

a *COVID infection and death rates pertain to the time period: 01/01/2020–02/07/2021*.

### CNAs' Training, Infection, and Mortality: Implications and Time for Action

COVID's dramatic exposure of both vulnerable SNFs' residents and CNAs who predominantly provide their daily care in SNFs' represents a threshold from which to learn from the past to better prepare for the future. Both healthcare providers and the general public realize now more than ever that not only is the U.S. (and world) not passed the full risks from COVID but also that future pandemics will recur (3). Devastating consequences from future pandemics will not recur, if proper preparation occurs. The timing is right for major change.

This policy statement underscores several problems with CNAs' training in need of further solutions. In terms of problems, CNAs, as the most regulated and trained direct care workers, still fall short of optimal regulations and training in general *and* in relation to the COVID pandemic based on multiple sources of secondary data. Infection prevention and control (IPC) is but one area in a larger training curriculum that emerges in practice as an add-on—largely a written one. That is, the law emphasizes having IPC systems in place but fails to get specific on CNAs' training in it (see SSA§ 483.80(a) (2)). If CNAs get trained, they may only receive a brochure and still satisfy regulatory requirements, because *how* training occurs pedagogically remains up to states' discretion (22, 23). Further, based on the wording of the regulations, this brochure may be received during continuing education classes and not during initial training, since the timing and mode of delivery is not mandated. Thus, the quality of the training on IPC varies per the statute leaving open the high risk for transmission to and/or mortality of vulnerable SNFs' residents. State variation in how they include IPC in training may only exacerbate the low quality of training. How COVID emerged in SNFs' and the ensuing “perfect storm” (3) suggest that a more centralized approach to CNA training in IPC must be implemented.

While the correlations between state-level training and COVID case rates among SNFs' residents in this policy brief did not indicate a statistically significant negative correlation, CNAs' training was statistically significantly negatively associated with SNFs' residents' mortality rates. Different factors affect mortality and case rates, which makes the varied results between them with CNAs' training less surprising (24–26). Differences between mortality and case rates' negative correlations with CNAs' training may be an artifact of the range of variation in the mortality and case data. While this possible methodological limitation in the data requires more controlled analyses, the data do indicate a linear correlation between CNAs' training and SNFs' residents' mortality rates. Further, this relationship with mortality held for both total and clinical hours suggesting that the distinction between clinical and total hours may be less than what was intended in the 1987 statute. In fact, clinical training is not “on the floor” training, because “on the floor” training occurs separately. Clinical training refers to the nature of the direct care (e.g., feeding, bathing, etc.) vs. indirect care (e.g., avoiding injuries, learning about dementia or communication, etc.) (27). CNAs need more “on the floor” training to refine what they learn in the classroom. COVID caused federal regulators to relax CNA training requirements to 8 hours online courses for “temporary” CNAs to accommodate staff shortages (28), which presents even more concern. However, this call to action is less about number of hours than it is about training content in IPC and its pedagogical effectiveness.

CNAs' training covers much more than IPC including many areas that may affect infection and mortality rates. However, these areas have shortcomings as well. For example, one area is communication with SNFs' residents including those living with dementia. In their initial training and possibly in the ACA's required “dementia care training,” CNAs read about “tips” for communicating with SNFs' residents (29), but communication is complex and dynamic especially for those SNFs' residents living with dementia. CNAs recognize the difficulty of caring for persons with dementia (22). Reading about communication will not reflect the reality of communicating, and CNAs cannot practice these tips until they are on the floor, if at all. Yet, communication represents another training area with implications for COVID infection and mortality rates. One reason some SNFs' did not give CNAs enough PPE had to do with scaring the residents (30). CNAs' training should include experiential learning of communication techniques embedded within strategies to emotionally connect with residents (14). Communication techniques and strategies would provide CNAs with concrete tools that they can use to tailor communication according to residents' individual needs. CNAs may have been better prepared to reduce fear among residents when residents saw PPE if CNAs had had effective communication training. Recommended competencies across a range of stakeholders related to the direct care workforce list communication in their top three (15).

Being able to emotionally connect with SNFs' residents using communication techniques may have potentially offset the social isolation that so many residents experienced after their families could no longer visit in person (31, 32). Understandably, the Nursing Home Reform Act of 1987 emphasized standards for task-oriented care for SNFs' residents (33), but as the COVID pandemic revealed, social health is as important as physical health. Sacrificing one at the other's expense can be deadly either way. If it becomes necessary to lockdown SNFs' in the future, CNAs may have to care for nursing home residents' physical and social health. Since neither of these training areas have received much emphasis to date, competency standards in both are direly needed (34).

Such an overhaul of CNAs' training following the new normal with COVID requires focused attention by experts–much the same way the Minimum Data Set (MDS) for SNFs' residents was revamped in the 2000s (24). Quality improvement in the SNFs' (and other long-term care settings for vulnerable older adults) has been disproportionately focused on SNFs' residents and far less so on the CNAs who provide an overwhelming amount of their daily care (35). Future efforts should balance out how to improve quality of life for both. In SNFs', given how long-stay residents *live* in the facilities, policymakers need to approach quality improvement for the CNA-resident dyads as opposed to individual residents. This approach requires an expanded mindset focusing on the relationships within the dyads.

Taking a dyadic mindset to quality improvement in SNFs' care requires not only additional emphasis on infection control and communication in the CNA training curriculum but also better data to monitor the impact of improved training (15). Currently, no federal requirements exist for SNFs' to collect data on the adequacy of CNAs' training initially or over time (22). The federally-required MDS includes quarterly data on residents' ability to communicate and to understand others, but no such data exist on CNAs' communication ability with the residents – self-reported or otherwise (36). Some data exist on this in the NNA survey from 2004, but more recent data need to be collected regularly similar to the MDS for residents. Census data, as presented previously, provides demographic data, but only an ongoing, nationally-based survey of CNAs would provide additional data on the quality of their training and work experience.

## Conclusions

**Figure 2:**
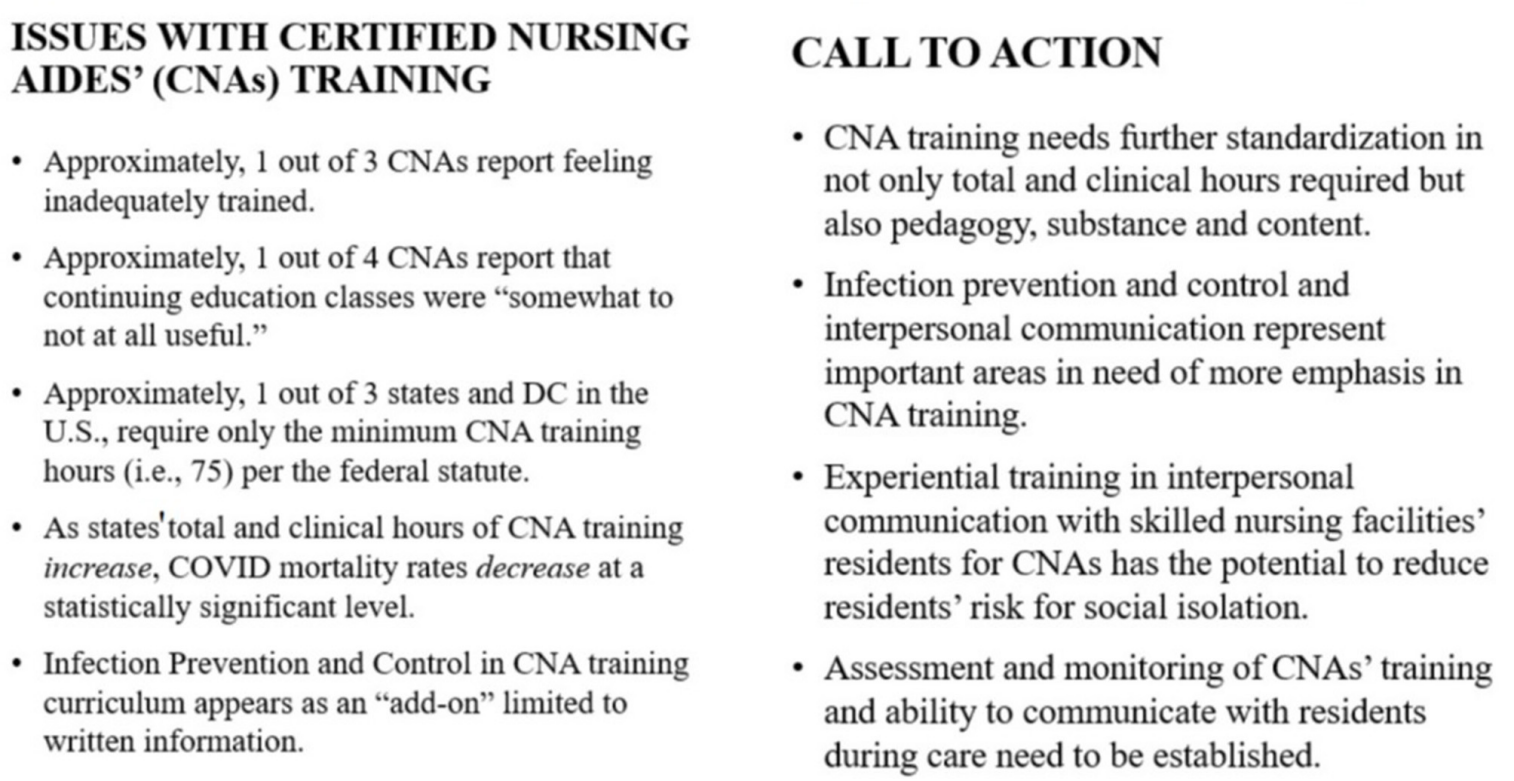
Summary of issues & Call to Action: Improvement in Certified Nursing Aides' Training.

Caring for SNFs' residents will continue to be in demand as the U.S. population continues to reach older ages disproportionately. In SNFs', the majority of the care is custodial and social in nature with assistance from CNAs for basic ADLs; yet, policies overwhelmingly emphasize medical/tasks vs. social aspects of care. COVID turned this emphasis upside down by causing vulnerable residents to be at risk both for increased mortality and social isolation simultaneously with little to no preparation for how CNAs needed to interact with the residents. The reality of providing care in SNFs' to vulnerable older adults and the current training standards and practices for CNAs to do so indicates a strong disconnect. As summarized in Figure 2, only through vastly improved training standards on content and pedagogy, experiential learning, and quality improvement monitoring for both CNAs and residents can the U.S. put health and social needs on the same level, even if only in SNFs' for the time being.

## Author Contributions

LL-S and AM-W both contributed to the conceptualization and design of the article, conducting data analysis, and writing the results. LL-S wrote the first full draft including the introduction, policy options, call to actions, and conclusion. Both authors contributed to the article and approved the submitted version.

## Funding

This work was supported by University of California, Los Angeles. LL-S received a Health and Aging Policy Fellowship (https://www.healthandagingpolicy.org/) funded by the Atlantic Philanthropies and the John A. Hartford Foundation in further support of this work.

## Conflict of Interest

The authors declare that the research was conducted in the absence of any commercial or financial relationships that could be construed as a potential conflict of interest.

## Publisher's Note

All claims expressed in this article are solely those of the authors and do not necessarily represent those of their affiliated organizations, or those of the publisher, the editors and the reviewers. Any product that may be evaluated in this article, or claim that may be made by its manufacturer, is not guaranteed or endorsed by the publisher.
